# From head micro-motions towards CSF dynamics and non-invasive intracranial pressure monitoring

**DOI:** 10.1038/s41598-021-93740-5

**Published:** 2021-07-12

**Authors:** Arnošt Mládek, Václav Gerla, Petr Šeba, Vladimír Kolář, Petr Skalický, Helen Whitley, Lenka Lhotská, Vladimír Beneš, Ondřej Bradáč

**Affiliations:** 1 Department of Neurosurgery and Neurooncology, 1St Faculty of Medicine, Charles University in Prague and Military University Hospital, Prague, Czech Republic; 2 Department of Cognitive Systems and Neurosciences, Czech Institute of Informatics, Robotics and Cybernetics, Czech Technical University, Prague, Czech Republic; 3 Department of Physics, University of Hradec Králové, Hradec Králové, Czech Republic; 4 Department of Technical Development, LINET Spol. S.R.O, Slaný, Czech Republic; 5 Department of Neurosurgery, 2nd Faculty of Medicine, Charles University in Prague and Motol University Hospital, Prague, Czech Republic; 6 Department of Natural Sciences, Faculty of Biomedical Engineering, Czech Technical University, Prague, Czech Republic

**Keywords:** Trauma, Brain injuries, Translational research

## Abstract

Continuous monitoring of the intracranial pressure (ICP) is essential in neurocritical care. There are a variety of ICP monitoring systems currently available, with the intraventricular fluid filled catheter transducer currently representing the “gold standard”. As the placement of catheters is associated with the attendant risk of infection, hematoma formation, and seizures, there is a need for a reliable, non-invasive alternative. In the present study we suggest a unique theoretical framework based on differential geometry invariants of cranial micro-motions with the potential for continuous non-invasive ICP monitoring in conservative traumatic brain injury (TBI) treatment. As a proof of this concept, we have developed a pillow with embedded mechanical sensors and collected an extensive dataset (> 550 h on 24 TBI coma patients) of cranial micro-motions and the reference intraparenchymal ICP. From the multidimensional pulsatile curve we calculated the first Cartan curvature and constructed a ”fingerprint” image (Cartan map) associated with the cerebrospinal fluid (CSF) dynamics. The Cartan map features maxima bands corresponding to a pressure wave reflection corresponding to a detectable skull tremble. We give evidence for a statistically significant and patient-independent correlation between skull micro-motions and ICP time derivative. Our unique differential geometry-based method yields a broader and global perspective on intracranial CSF dynamics compared to rather local catheter-based measurement and has the potential for wider applications.

## Introduction

Raised intracranial pressure (ICP) is a critical problem in neurosurgical and neurological practice. It can arise as a consequence of traumatic brain injury (TBI), intracranial lesions, disorders of cerebrospinal fluid (CSF) circulation and more diffuse intracranial pathological processes. Monitoring and treating ICP has major influences on outcomes, allowing early detection of secondary damage and rapid therapeutic intervention. However, the gold standard of measurement involves invasive craniotomy and insertion of a catheter, a procedure with known associated risks such as infection, hemorrhage, and tissue lesions. Therefore, there is a need for a non-invasive means of measuring this critical parameter.

Each of the available ICP assessment modalities has limitations when compared to the gold standard. Canac et al.^1^ group these modalities into: ophthalmic, otic, fluid dynamic, and electrophysiologic.

The optic nerve sheath expands with increasing ICP^2,3,4^ and the best measure of optic nerve sheath expansion is ultrasound (sensitivity 90%; 95% CI 87–92, specificity 88%; 95% CI, 84–91)^3^. However, studies of ICP association are inconclusive^5^: some find a strong positive correlation^6^; others suggest the parameter unreliable^7,8^. Venous ophthalmodynamometry uses the principle that increased ICP affects the central retinal vein^9^, but changes may be masked by vascular disease. Optical coherence tomography obtains high resolution retinal images (increased ICP causes retinal nerve fiber swelling) but is unreliable in high levels of papilledema^10^. Pupillometry can detect ICP changes^11^, but ICP values can not be derived. This remains a screening rather than a monitoring tool^5,12^.

Otic measurements utilize the principle of continuous CSF and perilymphatic spaces. Infrasonic emissions from the tympanic membrane can be continuously monitored^13^, opening the possibility to establish ICP values. Otoacoustic emissions generated by perilymph oscillations correlate with ICP changes^14,15^, but these studies are too small for clinical use^5^. Acoustoelastic properties of ultrasound were used to measure ICP in an experiment modeled on mechanical properties of the brain and skull, but animal or clinical studies have not been conducted^15^.

Transcranial doppler can estimate ICP^16–18^, but without a standardized formula, derived ICP values will vary^19^. Tissue resonance analysis uses the principle that each organ resonates at a unique frequency, with ICP related to brain tissue resonance^20^. Using the third ventricle as an echo chamber, one study obtained an echopulsogram representing ICP waves^20^—a promising technique needing further study^5^. Two-depth ophthalmic artery Doppler invented by Ragauskas et al.^21,22^ derives ICP from flow velocities, but clinical validation showed inappropriately wide Bland–Altman 95% limits^23^. The Rotterdam transducer monitors flow via the anterior fontanell but is only useful for infantile hydrocephalus^24^.

Electroencephalogram studies show signature alterations preceding ICP increase^25^, but some correlations were weak^25–27^. Near infrared spectroscopy detects changes in cerebral bloodflow^28^, one study showing clear signal changes accompanying ICP changes^29^ but was not designed to assess correlation with ICP^5,11^. Alterations in latency of visual evoked potentials are linked with ICP but this method does not allow continuous monitoring, and issues with variability in latency, amplitude, and waveforms remain^30^.

## Methods

### Theory

During systole blood is pushed into the cranial cavity. The blood stream represented by a pressure wave (pulse) propagating upwards along the arteries is associated with a momentum which, according to the momentum conservation principle, can only be transformed into successive physical processes, cranium expansion being one of them. Following the branching arteries, the blood gets closer to the cranial vault and is partially reflected on the inner bone surface. The momentum of the reflected blood is relayed to the skull leading to subtle head tremble that can in principle be detected. Since the artery branching is not fully symmetric with respect to the body axis, the head tremble is a very complex process and summarizes all the particular pulse reflections inside the skull.

In our approach we use an in-house built pillow placed under the head and equipped with imbedded sensitive mechanical sensors. The pillow measures projections of the head motions in directions perpendicular to the individual sensors. As a result, what we obtain from the pillow is the head tremble as seen from the coordinate system related to the sensors.

The physical processes inside the skull can be represented by a multi-dimensional curve β, a geometrical object that describes the head tremble globally. Each sensor provides a one-dimensional projection of the head tremble and represents a respective component of β in a particular direction. While displacement of the sensors with respect to the head inherently changes the individual β components, the β curve alone does not change, since the pillow reorientation has no effect on the in-skull processes, we just observe the resulting head tremble from a different angle. Simply put, the multi-dimensional β curve constitutes a "fingerprint" of the intracranial processes from which CSF dynamics can be inferred.

According to differential geometry, for a given smooth *n*-dimensional curve β there are exactly *n-1* functions k_1_(t), k_2_(t), …, k_n-1_(t) that completely determine the curve. These functions are denoted as Cartan curvatures and can be evaluated from the measured one-dimensional signal projections. Once obtained, the k(t) functions are Euclidean invariants, i.e. do not change with the head motion despite being calculated from single head position-dependent projections. In the present study we restrict ourselves to the k_1_(t) term only due to the following reasons: (i) k_1_(t) is evaluated using only the second derivatives of the signal and is therefore not very sensitive to the signal background noise. At the same time, it contains enough information about the investigated process, and (ii) calculation of the higher k_n_(t) terms demand *n* + *1* derivatives of the signal are hence more sensitive to the noise. For further details, see underlying theory paragraph in the Supplementary Information.

### Measurement set-up

**Figure 1:**
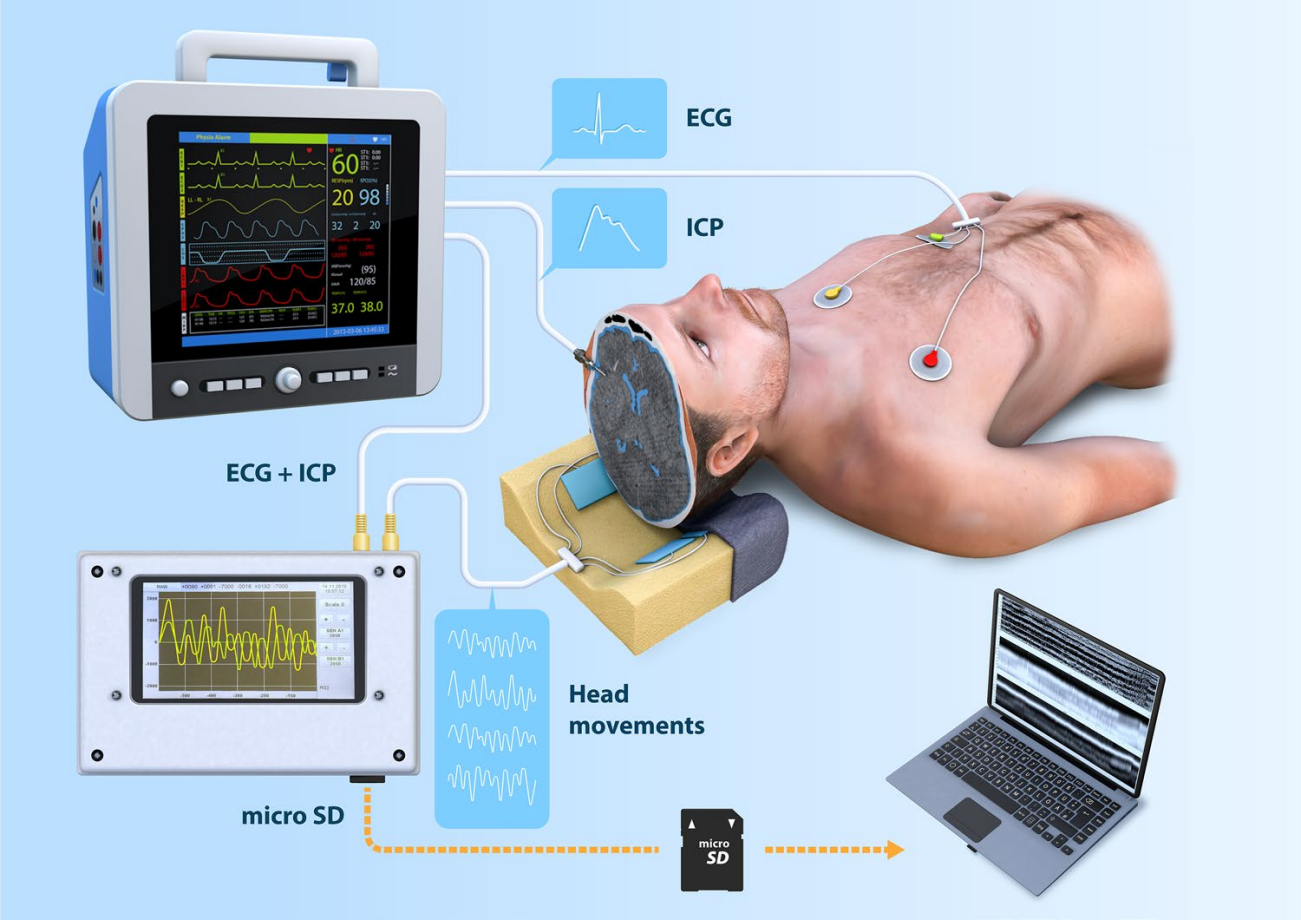
The measurement set-up. The head micro-motions were detected using four pillow-embedded sensors. Along with the ECG and ICP signals acquired through a bedside monitor, the time series were recorded on-the-fly using an in-house built logger. Please note that the figure is only for illustrative purpose and does not reflect the actual measurement setting, e. g. in the actual setting the patient’s head was elevated by 40° throughout the measurement. The figure was generated using Adobe Photoshop 2020, Adobe Illustrator 2020 (both https://www.adobe.com/) and Cinema 4D, version R20 (https://www.cinema4d.cz/).

The measurement hardware set-up is illustrated in Fig. 1. The following six signals were synchronously monitored and recorded on-the-fly for post-processing: (i) ECG, (ii) reference ICP measured via parenchymal catheter, and (iii) mechanical signals of head tremble registered by four pillow-embedded pressure sensors. ECG and ICP signals were acquired from the bedside monitor analog output and together with the mechanical signals recorded on a memory card at high temporal resolution (1 kHz) using our in-house built electronic logger. The analog signals were internally digitized by 12-bit ADC.

### Signal analysis

**Figure 2:**
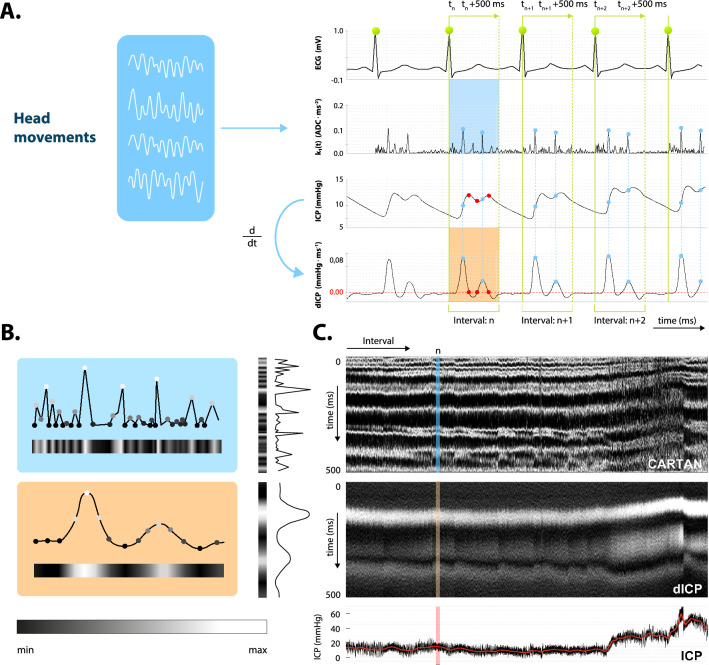
(**A**) The analysis starts with the pre-processing of the six synchronous signals: the four mechanical signals from the pillow-embedded sensors (ADC units), ECG (mV) and ICP (mmHg). The four-dimensional mechanical β curve is then represented by the one-dimensional k_1_(t) curvature invariant (ADC∙ms^−2^). The raw ICP signal is smoothed and time derived yielding dICP (mmHg∙ms^-1^). The k_1_(t) and dICP signals are fragmented according to the ECG R-peaks (green vertical lines) into elementary intervals (EI). From each EI only the first 500 ms are considered. (**B**) Color coding of k_1_(t) (blue box) and dICP (orange box). **C**. The Cartan (top) and dICP (middle) map after merging rotated strips side-by-side from left to right; the n-th EI is highlighted in blue and orange, respectively. Bottom: evolution of ICP average value (mmHg). The figure was generated using Adobe Illustrator 2020 (https://www.adobe.com/).

The process of signal analysis is illustrated in Fig. 2. From the mechanical signals represented by a four-dimensional β curve we compute the one-dimensional first Cartan curvature k_1_(t) (ADC∙ms^-2^, where ADC stands for analog-to-digital converter units), which is non-negative and features numerous peaks of different magnitudes. Each peak corresponds to a single head motion detected at a specific time, while the magnitude gives evidence about the rate of change of the β curve.

A typical ICP waveform contains three extrema denoted P1—P3. Even though a lot of attention is given to the interpretation of these extrema (Fig. 2A, red dots) and their relation to the ICP (mmHg) average value, from our perspective instants of ICP time derivative (dICP (mmHg∙ms^-1^)) extrema are more relevant (Fig. 2A, blue dots) since these are the moments when CSF dynamics peaks and the pressure wave hits the skull. Note that at P1—P3 dICP equals zero, indicative of a momentary equilibrium state with no net CSF flux.

CSF dynamics are tightly coupled to the cardiac cycle. In the study we adopt the time-locking procedure in which both k_1_(t) and dICP signals are segmented based on the ECG (mV) into so-called elementary intervals (EI). The n-th EI begins at the time of the n-th systolic R-wave t_n_ and ends at t_n_ + 500 ms (Fig. 2A, solid and dashed green vertical lines, respectively).

The Cartan k_1_(t) and dICP signals are fragmented into N 500 ms long EIs. The numerical values of k_1_(t) and dICP within each interval are normalized to 1 and mapped via gray scale (black: 0, white: 1) to a strip (Fig. 2B, k1(t): blue rectangle, dICP: orange rectangle). The strips are then rotated 90° in clockwise direction and merged horizontally side-by-side from left to right. What we obtain is the Cartan and dICP map (Fig. 2C) with x-axis representing the EI index n ∈ {0, 1, …, N} and y-axis representing the relative time within the interval t ∈ (0, 500) ms. The Cartan maps exhibit a number of white maxima bands each of which is associated to some mechanical event occurring approximately at the same time in every cardiac cycle. The dICP maps display a lower number of maxima bands with the first one being the most prominent. Please note that since we deal only with the k_1_(t) and dICP relative maxima time shifts with respect to the respective R-wave there is no need—for the purpose of the present study and at the current stage—to calibrate the method. An initial calibration will be, however, necessary should the absolute value of the intracranial pressure be evaluated. Theoretically this is possible because both the knowledge of the time shift of dICP is known (i.e. the maximum of k_1_(t)) and the pulse arrival time of the radial artery is known, as it is directly measured by a cannula needle. The length of the radial artery can be derived from basic anthropometric data, and other factors that might alter the pulse arrival time include anatomic variations, disease states, differences between mechanical properties of the arteries (muscular vs. elastic), pharmacologically altered smooth muscle tone, etc. These technical details render the calibration process a non-trivial task, beyond the scope of the present study.

### Clinical overview of tested subjects

A total number of 24 patients (P01–P24) were included in the study; 21 males and 3 females with an average age of 47.3 ± 18.5 (Table 1). The admission GCS status ranged between 3—15 with the median score of 3 and IQR of 3 (Q_3_ = 6, Q_1_ = 3). These patients were admitted to the Emergency department of the Military University Hospital, Prague with suspected TBI alone or as a part of polytrauma. All patients underwent triage, clinical examination, laboratory testing, abdominal ultrasound and whole-body CT including CT angiography of the brain. After the initial triage and treatment of any immediate life-threatening conditions, patients were placed in the ICU for further management. All these patients had initial and subsequent neurological examination and brain CT. The Marshall CT-based classification of TBI was used to predict the patient’s outcome (Table 1). 13 patients suffered diffuse injury II, 7 diffuse injury III with swelling, 2 had evacuated mass lesion V and 2 had non-evacuated mass lesion VI. Patients with signs suggestive of elevated ICP but without indication for immediate surgery were managed with intraparenchymal ICP monitoring. The average initial ICP obtained immediately after catheter insertion was 15.5 ± 9.0 mmHg. An ICP monitor was placed preferentially through the right prefrontal cortex, except for in cases where it was necessary to place it through the left (localized expansions—contusion, hemorrhage, ischemia or known right hemisphere dominance). After the operation, a pillow equipped with mechanical sensors was placed under the head of the patient. This included a connection to a bedside monitor (Fig. 1).

**Table 1:** Overview and admission status of the measured TBI patients (P01–P24): Marshall CT classification score, Glasgow Coma Scale (GCS) and the initial ICP (mmHg) after catheter insertion.

Patient ID	Sex	Age	Marshall score	Admission GCS	Initial ICP (mmHg)
P01	M	29	II	3	16
P02	F	67	III	3	21
P03	M	32	II	15	15
P04	F	66	III	4	15
P05	M	68	III	3	15
P06	M	70	II	3	12
P07	M	31	III	3	17
P08	M	36	V	3	20
P09	F	39	III	3	29
P10	M	21	II	7	24
P11	M	75	V	3	2
P12	M	66	II	6	10
P13	M	44	II	3	26
P14	M	33	II	3	10
P15	M	33	II	6	2
P16	M	41	II	10	18
P17	M	31	III	3	17
P18	M	79	VI	3	12
P19	M	33	II	6	2
P20	M	27	III	3	40
P21	M	41	II	14	2
P22	M	58	VI	3	15
P23	M	40	II	3	20
P24	M	76	II	14	13

Elevated intracranial pressure was treated with respect to current evidence^31–36^. Pharmacological interventions included continual or bolus intravenous application of hypertonic sodium solutions or mannitol with a preference towards hypertonic solutions due to a decreased risk (risk ratio [RR] = 0.39; 95% CI = 0.18–0.81) of ICP treatment failure^31^, continual intravenous opioids and sedatives. In cases of refractory ICP elevation, barbiturate coma was induced by an initial loading dose followed by continuous application. Non-pharmacological interventions included temporary hyperventilation with a target PaCO_2_ of 30 to 35 mmHg as a bridge to definitive management and nursing care including appropriate positioning (40° head elevation). Patients with refractory elevated ICP where no more conservative options were possible, or brain CT was suggestive for surgical management underwent surgical intervention. In the case of decompressive hemicraniectomy the pillow-based head tremble monitoring was terminated. Therefore, all mechanical data presented in this study were acquired prior to decompressive hemicraniectomy.

### Correlation analysis

**Figure 3:**
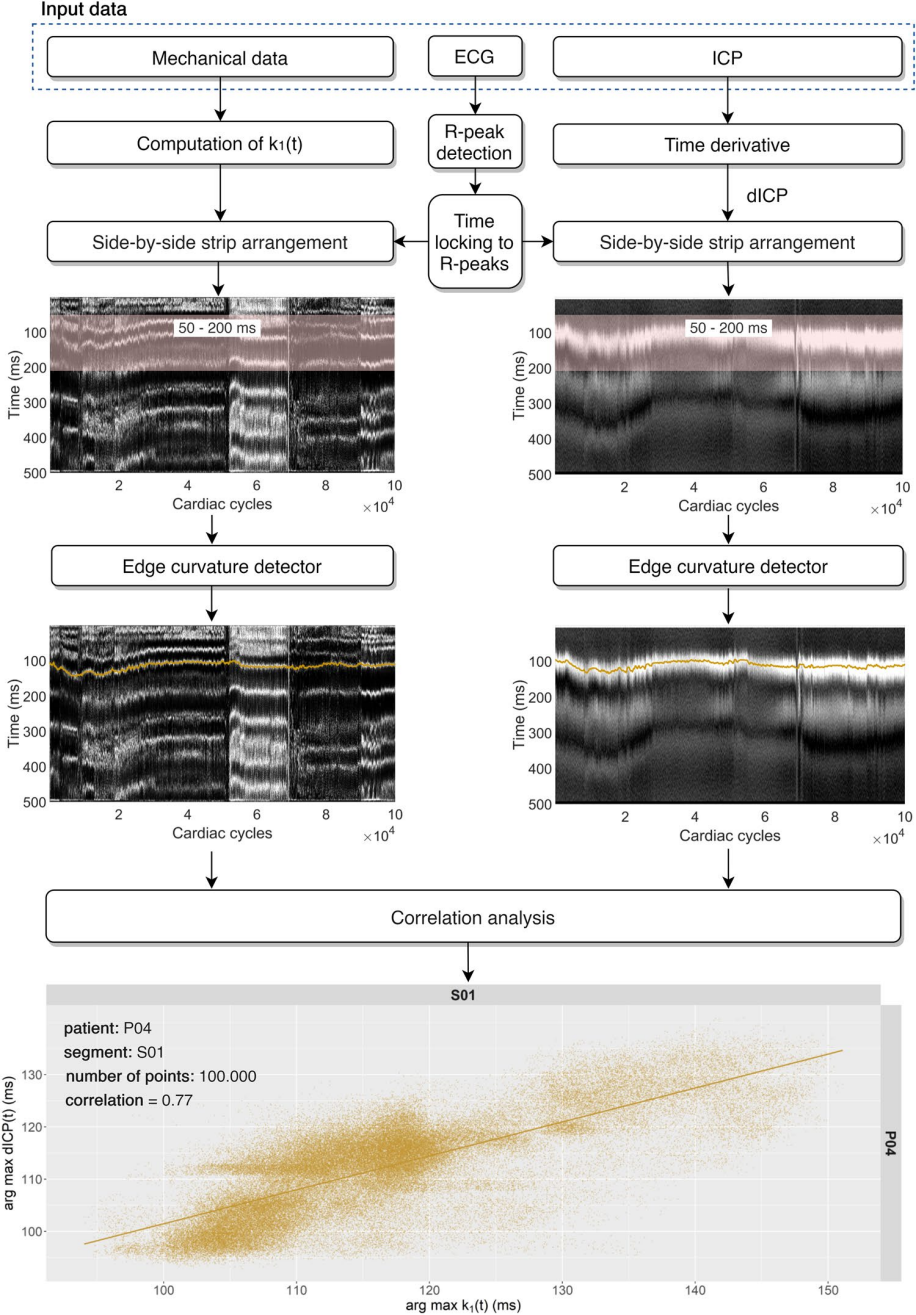
Block diagram of the signal processing methodology. These data refer to the patient P04 and the segment S01. The total number of points in the correlation plot (EIs) is 100,000; the Pearson's correlation coefficient between the detected edge curvatures (orange lines) is 0.77. The plots were generated using Matlab2020a (Cartan maps, https://www.mathworks.com/), R 4.0.0 (R Core Team (2020). R: A language and environment for statistical computing. R Foundation for Statistical Computing, Vienna, Austria. URL https://www.R-project.org/.) with ggplot2 3.3.2 (H. Wickham. ggplot2: Elegant Graphics for Data Analysis. Springer-Verlag New York, 2016.) and the figure was assembled using diagrams.net on-line tool (https://www.diagrams.net/).

The process of correlation analysis is shown as a flowchart in Fig. 3. The Cartan k_1_(t) curvature is computed from mechanical data; dICP is computed as the time derivative of the ICP signal. Time-locking ECG-based segmentation and the side-by-side arrangement of the EI strips yield the Cartan and dICP maps. The edge curvatures (white maxima bands) are semi-automatically detected for both Cartan and dICP maps (Fig. 3, orange lines). Finally, the correlation analysis is applied to the selected k_1_(t)-dICP pairs of the edge curvatures.

## Results

We obtained a total of approximately 574 h of recorded signals from 24 ICU coma patients (Table 2). Data were manually examined and analyzed using an in-house built MATLAB code designed to seek out the longest uninterrupted segments with the minimum threshold length of 5,000 consecutive elementary intervals (EIs). The duration of the shortest segment composed of 5,000 EIs was approximately 1.2 h for an average heart rate of 70 bpm. The number of continuous segments found in each patient's record ranged between 1 (P11, P13, P17, P23) and 11 (P16). The average number of segments per patient was 4.5. The total number of segments for all patients was 108 and the mean segment length was over 100,000 EIs (approximately 24 h of uninterrupted continuous data). For each segment, the respective Cartan and dICP map was calculated and subjected to the semi-automated edge detector algorithm.

**Table 2:** List of measured patients (P01–P24) and analysis results.

Patient ID	#Seg	Used #EI	Total #EI	Util. (%)	Time (hours)	Correlation coefficient	Linear slope
P01	4	45,000	203,522	22.1	10.7	0.73 ± 0.09	0.63 ± 0.40
P02	3	44,000	234,518	18.8	10.5	0.71 ± 0.01	0.69 ± 0.14
P03	10	180,000	714,118	25.2	42.9	0.74 ± 0.10	0.95 ± 0.27
P04	3	220,000	531,888	41.4	52.4	0.76 ± 0.02	1.25 ± 0.60
P05	9	273,000	511,853	53.4	65.1	0.76 ± 0.11	0.93 ± 0.08
P06	8	212,000	779,019	27.2	50.5	0.76 ± 0.09	0.81 ± 0.18
P07	5	156,000	432,682	36.1	37.1	0.72 ± 0.09	0.73 ± 0.25
P08	9	236,000	944,134	25.0	56.2	0.76 ± 0.06	0.62 ± 0.16
P09	2	84,000	136,773	61.4	20.2	0.72 ± 0.16	0.79 ± 0.63
P10	5	80,000	474,700	16.9	19.0	0.76 ± 0.03	1.20 ± 0.21
P11	1	10,000	495,318	2.0	2.4	0.64 ± 0.00	0.86 ± 0.00
P12	2	20,000	362,159	5.5	4.8	0.66 ± 0.06	0.75 ± 0.20
P13	1	6,270	18,770	33.4	1.5	0.81 ± 0.00	1.16 ± 0.00
P14	10	140,300	433,607	32.4	33.4	0.74 ± 0.09	0.90 ± 0.21
P15	4	46,500	203,512	22.8	11.1	0.80 ± 0.06	0.79 ± 0.12
P16	11	216,000	405,808	53.2	51.4	0.74 ± 0.11	0.89 ± 0.26
P17	1	56,836	56,836	100.0	13.5	0.89 ± 0.00	0.94 ± 0.00
P18	5	84,000	320,603	26.2	20.0	0.75 ± 0.07	0.79 ± 0.09
P19	2	30,000	275,091	10.9	7.1	0.66 ± 0.04	0.44 ± 0.06
P20	3	90,000	463,417	19.4	21.4	0.81 ± 0.15	0.90 ± 0.20
P21	2	20,000	338,910	5.9	4.8	0.73 ± 0.12	1.26 ± 0.58
P22	2	40,000	505,968	7.9	9.5	0.71 ± 0.01	0.76 ± 0.21
P23	1	10,000	431,123	2.3	2.4	0.71 ± 0.00	0.78 ± 0.00
P24	5	111,000	141,156	78.6	26.4	0.71 ± 0.03	0.92 ± 0.07
Total	108	2,411,206	9,415,485	NA	574.1	0.86	0.68
Average	4.5	100,467	392,312	25.6	23.9	0.74 ± 0.08	0.86 ± 0.27

#Seg.: number of used continuous segments, each segment is composed of at least 5 000 sequential EIs; Used #EI: total number of EIs summed over used segments; Total #EI: total number of all recorded EIs; Util.: Used #EI/Total #EI ratio in %; Time: approximate duration (hours) of used segments for average heart rate of 70 bpm; Corr.Coef.: Pearson's correlation coefficient between selected k_1_(t) and dICP maxima bands in used segments; Lin.Slope: linear regression slope in used segments.

In the dICP maps up to three maxima bands can be recognized with the first one being the most prominent and well-defined. The first dICP maximum band can be detected within the range of 60–180 ms in each EI and is associated with the highest rate of ICP elevation towards the P1 peak. In line with the higher variability of the occurrence of P2 and P3 peaks in the ICP signal, their associated dICP maxima bands are less pronounced and often blurry. These bands are therefore less useful in analysis due to the high level of noise (Fig. 3). The number and layout of the Cartan map maxima is heterogeneous and patient-dependent compared to the dICP map, yet there are several patterns that seem to be universal. In each patient we were able to identify a k_1_(t) maximum band within the range of (t_n_ + 50 ms, t_n_ + 200 ms), which coincides in terms of shape and onset time with the first dICP maximum.

**Figure 4:**
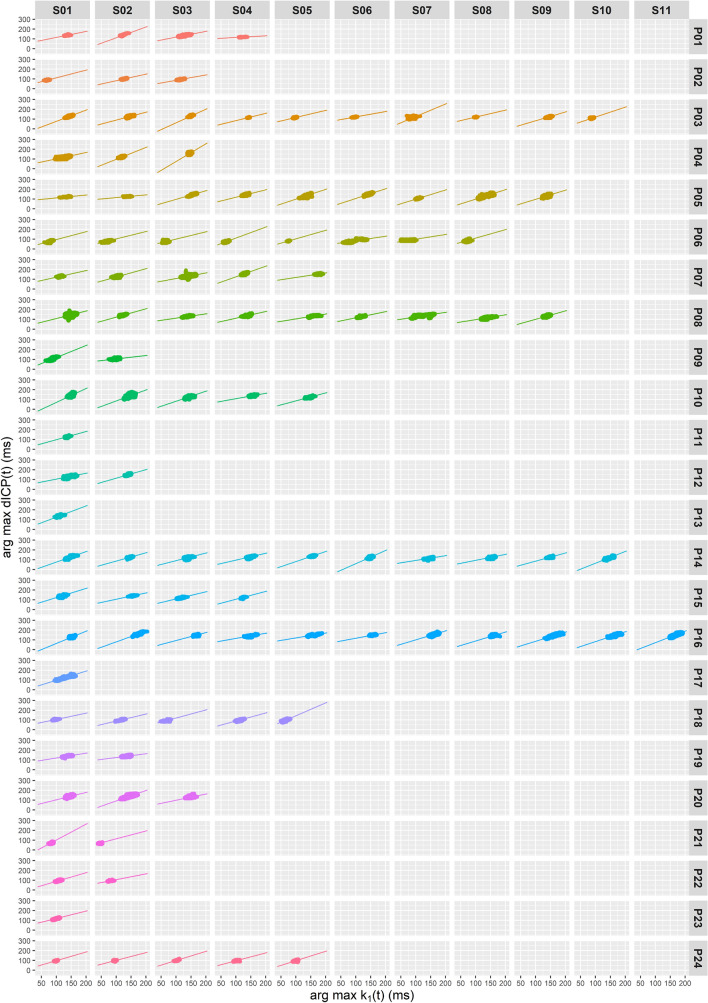
Correlation analysis for individual segments. Correlation between the selected k_1_(t) and the first dICP maximum (ms) drawn separately for all 108 segments. The figure was generated using R 4.0.0 (R Core Team (2020). R: A language and environment for statistical computing. R Foundation for Statistical Computing, Vienna, Austria. URL https://www.R-project.org/.) with ggplot2 3.3.2 (H. Wickham. ggplot2: Elegant Graphics for Data Analysis. Springer-Verlag New York, 2016.).

Using the semi-automatic edge curve detector algorithm, the curves connecting respective k_1_(t) and the first dICP maxima were calculated separately for each segment. The Pearson's correlation coefficient between the time of the selected k_1_(t) and the first dICP maxima within each of the 108 segments was between 0.62 (P12/S02) and 0.94 (P05/S08) with the mean correlation coefficient averaged over all segments being 0.74 ± 0.08 (Table 1). Additionally, we performed linear regression analysis. The mean linear fit slope averaged over all segments was 0.86 ± 0.27. The value of the average linear slope lower than 1.0 is in line with our expectations reflecting the cause: the inbound ICP wave hitting the cranial vault and the resulting effect—a detected head tremble. It should be noted, however, that in patients P04, P10, P13 and P21 the average slope is slightly higher than 1.0. The reason remains unclear and is likely associated with inferior data quality. The correlation between k_1_(t) and dICP maxima bands in each segment is illustrated in Fig. 4. One important point must be stressed here: during the invasive ICP measurement the position of the tip of the measuring sonde with respect to the skull is patient dependent. The measuring tip is placed inside the brain in a small but not negligible distance from the skull bone. Hence there is a small, but not negligible time shift in the pulse arriving to the measuring tip and to the skull bone. Moreover, the invasive sonde measures ICP locally at the point it has been inserted to, whereas the head tremble is the common result of all pulse reflections inside the skull.

**Figure 5:**
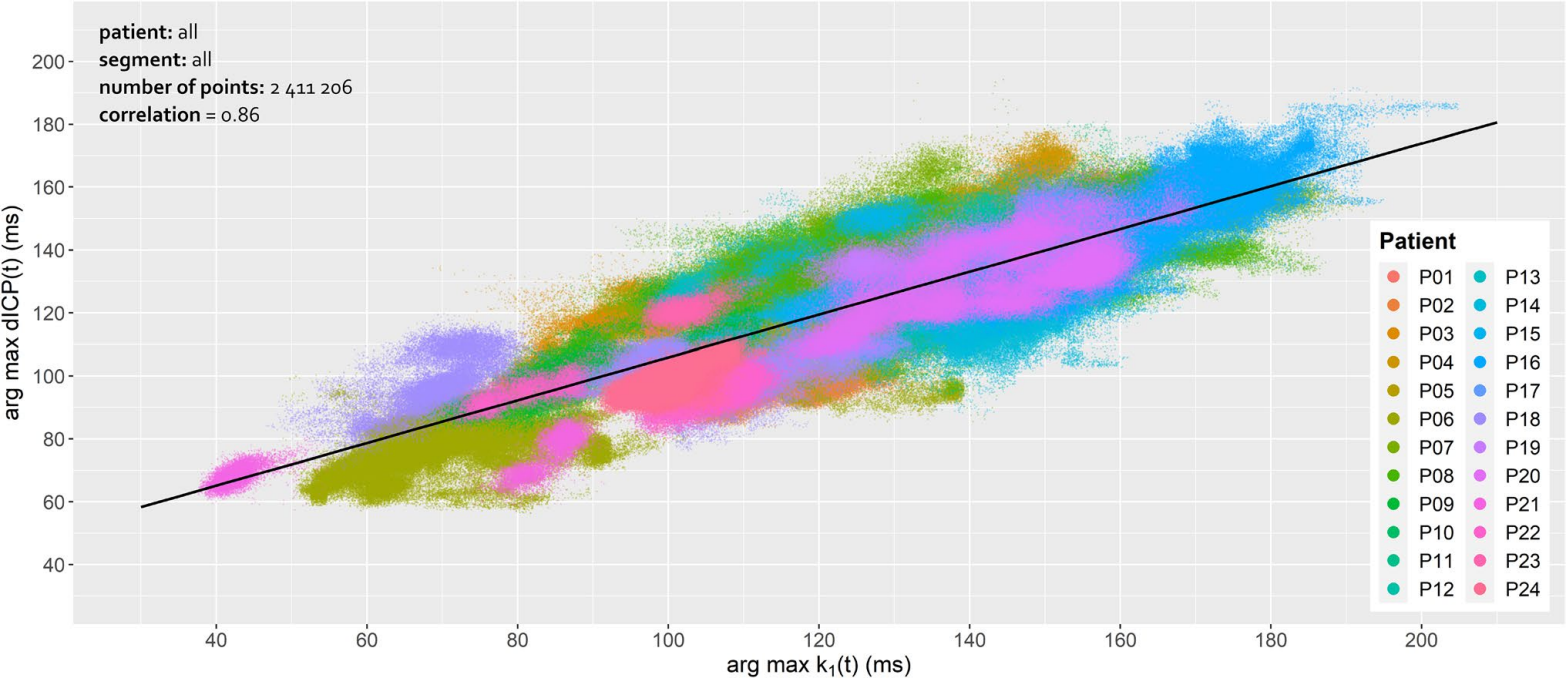
Correlation analysis for all segments. Correlation between the selected k_1_(t) and the first dICP maximum (ms) drawn for all 24 patients. The figure was generated using R 4.0.0 (R Core Team (2020). R: A language and environment for statistical computing. R Foundation for Statistical Computing, Vienna, Austria. URL https://www.R-project.org/.) with ggplot2 3.3.2 (H. Wickham. ggplot2: Elegant Graphics for Data Analysis. Springer-Verlag New York, 2016.).

The slope of the k_1_(t)-dICP linear correlation depends on a number of internal (brain parenchyma and skull properties) and external (pillow orientation with respect to the patient's head) parameters. Similarly, the time of the first dICP maximum following systole depends on various physiological quantities such as the elasticity of arteries and can range by tens of milliseconds. To investigate patient-independent correlation between k_1_(t) and dICP maxima we merged data from all patients (approximately 2.4⋅10^6^ of EIs). The correlation coefficient between the time of the selected k_1_(t) and the first dICP maxima calculated for all patients and segments was 0.86, and the slope value was 0.68 (Table 1 and Fig. 5).

## Discussion

ICP monitoring is a vital part of management of pathological conditions that can result in an elevation of ICP, for example TBI and the associated pathophysiological mechanisms (diffuse axonal injury, diffuse or focal cerebral edema, intracranial hemorrhage, or contusions with mass effect, etc.). ICP monitoring is also important in other situations such as non-traumatic intracranial hemorrhage or less commonly in large-vessel ischemic stroke. Currently, the Brain Trauma Foundation 4th edition guidelines provide a Level IIB recommendation for the use of ICP monitoring in the management of patients with severe TBI to reduce in-hospital and 2-week postinjury mortality^37^.

Given the generally known principles of Monro-Kellie doctrine, the compensatory mechanisms for further additions in the intracranial volume are limited. Once the compensatory mechanisms are exhausted ICP substantially rises and compression of internal structures occurs, causing secondary brain damage, ischemia, and infarction. In acute brain injury, the relationship between cerebral blood flow (CBF) and cerebral perfusion pressure (CPP) is altered. The ability to autoregulate is lost, and CBF becomes linearly passive to CPP. CPP values are mathematically dependent on ICP via the well-known equation CPP = MAP—ICP, where MAP is the mean arterial pressure. Therefore, to be able to minimize secondary brain damage, knowledge of the exact ICP values is critical^35^.

Management of intracranial hypertension depends on the underlying cause. Stage 1 interventions are head elevation, sedation, and analgesia. If medical management is insufficient, surgical options, such as drainage of cerebrospinal fluid or evacuation of mass lesions, are considered. In the absence of surgical options, stage 2 treatments are initiated, consisting of either mannitol or hypertonic saline and hyperventilation. Stage 3 therapies include hypothermia, metabolic suppression (barbiturates), or craniectomy as a definitive option^35^.

The gold standard for ICP monitoring is the placement of invasive ventricular or intraparenchymal ICP monitor^38–40^. Due to the risk of potential complications (infection, post-procedural hemorrhage or catheter misplacement) associated with invasive ICP monitoring, various non-invasive modalities have been suggested, and as of yet none can fully substitute for invasive monitoring^40,41^.

Visualization of differential geometry invariants of multi-dimensional mechanical signals in concert with ECG time-locking represents a unique way to gain deeper insight into intracranial physical processes. From the theoretical point of view each k_1_(t) maximum corresponds to some mechanical event whose manifestation through a pressure change is recognized by sensors. To distinguish somatic events coupled to the cardiac cycle from the stochastic perturbations contaminating the signal, we utilized an ECG time-locking method for segmenting k_1_(t) into EIs, which, when merged side-by-side yield aso-called Cartan map. The great asset of displaying results via maps is that the physiological cardiac cycle-dependent processes appear as white apparent maxima bands, while the cardiac cycle-independent noise interfering with the signal appear as scattered white points.

In contrast to dICP, Cartan maps exhibit several maxima bands across the whole 500 ms interval. Each regular maximum is related to some recurrent process emerging at a similar time after the systole onset. However, there is still considerable controversy surrounding the assignment of each maximum band to the underlying physical process. Besides, the total number of detected maxima appears to be patient-dependent, potentially indicating anatomical variations. Even though further research is required to fully comprehend and relate recurrent maxima to the respective physiological processes, there are several apparent patterns that are common in all patients. We are of the opinion that it is reasonable to restrict to a (t_n_ + 50 ms, t_n_ + 200 ms) sub-interval due to the following reason: the k_1_(t) maxima occurring before t_n_ + 50 ms are unlikely to be linked with any intracranial process as it is too early for the arterial pressure wave to reach the cranial cavity. Instead, these premature maxima can likely be ascribed to the isovolumetric heart muscle contraction, aortic valve opening and the blood ejection into the aortal arch or a possible reflection on the carotid sinus. Moreover, there are an indefinite number of indistinguishable superimposed maxima bands before t_n_ + 50 ms which supports our assumption of persisting noise and supports the fact that the early hemodynamical processes in the chest are not always visible in the head motion. Rather, the phenomena depend on the patient’s position, skeleton, musculature etc. A similar situation arises after t_n_ + 200 ms. The respective late k_1_(t) maxima are often blurred, poorly separated and difficult to track. It seems that the fuzzy appearance of the Cartan map after t_n_ + 200 ms results from the superposition of CSF dynamics with various parasitic processes such as secondary arterial pressure waves reflected on large arterial bifurcations in the lower part of the body, and vibrations transferred to the head via spinal cord. For the arterial reflection process see the seminal work of O’Rourke^42^.

Our findings appear to be well substantiated by the fact that the sensors detect dynamic pressure changes (more precisely the change of the pressure projection perpendicular the sensor surface). The imperceptible expansion of the calvaria follows the rapid elevation of ICP which is most dominant in the pre-P1 period. This is also the time period during which the mechanical impulse of the injected blood pulse must be transcribed into the head motion due to the basic Newtonian conservation laws. We are therefore confident that this particular k_1_(t) maximum refers to the mechanical response of the cranium vault to the rising ICP.

We believe that the measured mechanical signal originates from the pressure wave propagated via the intracranial arteries, while the mechanical impulse transferred by the extracranial arteries does not contribute because of a difference in the artery histology. To be exact: the common carotid artery is elastic, i.e. has a denser elastic lamina, a thinner media with a small development of elastic fibers, and lacks external elastic lamina. However its intracranial and extracranial branches are muscular. As soon as the internal carotid artery enters the skull its intimal medial thickness, i.e. the total number of smooth muscle fibers and of the elastic fibers is significantly reduced, and it becomes similar to an elastic artery. For further details see the work of Aggarwal et al.^43^.This implies that the intracranial and extracranial arteries display different blood pulse propagation. Moreover, roughly 65% of the blood pulse propagates via the common carotid artery to the internal carotid artery and only 35% contributes via the external carotid artery to the extracranial head circulation^44^. The pulse reflection on muscular arteries is well understood and appears on their subsequent branching into the smaller arteries, finally being stopped in the arterioles. The head itself is roughly left/right symmetric. This means that the mechanical impulse transferred by the extracranial circulation is summarized along the body axis and is tangential to the sensors. The extracranial circulation will thus be hardly visible by the sensors and cannot substantially interfere with the signal obtained from the intracranial blood pulse.

One issue that remains to be dealt with is the susceptibility of the mechanical signal to external perturbations. The quality of the mechanical data recorded by the highly sensitive pressure sensors can easily be compromised due to the interference with the vibrating mechanical pulmonary ventilation slightly lifting patient's head, suboptimal orientation of the patient's head with respect to the pillow leading to an unevenly distributed load on the sensors or repositioning of the patient by the ICU staff. It should be noted, however, that the movement of the patient by the staff alters the measurement of the invasive ICP as well and that the transient reduction of the signal quality does not diminish the clear clinical asset in monitoring ICP evolution on the minutes to hours timescale.

Although not demonstrated in the present work, we have strong indications that the dICP maximum time (and hence k_1_(t)) linearly anti-correlates with the mean ICP value (manuscript in preparation, see Fig. 2C) so when ICP rises, the first dICP maximum appears earlier after the onset of systole. According to our hypothesis the higher ICP compresses the brain arteries and makes their walls effectively stiffer. As a result, propagation of the pressure wave is more rapid, and we observe is a slight negative time shift of the dICP maximum. Similar time shifting in the same direction can be seen in the second and third dICP maxima which seem to be even more sensitive to the ICP change. Even though further discussion on the dICP-ICP correlation extends beyond the present work we believe that the average ICP value affects dICP morphology, which is followed by skull micro-motion globally reflected in Cartan maps. Therefore, ICP dynamics can in principle be followed by monitoring skull motions and their relative time positions within the cardiac cycle.

## Supplementary Information

Supplementary Information.

## Data Availability

The datasets analyzed during the current study are available from the corresponding author on reasonable request.
