# Supplementary material for: From head micro-motions towards CSF dynamics and non-invasive intracranial pressure monitoring
Source: Sci Rep. 2021 Jul 12;11:14349. doi: 10.1038/s41598-021-93740-5 (PMC8275772; doi:10.1038/s41598-021-93740-5)
Supplement: Supplementary file 1 — Supplementary Information. [file 41598_2021_93740_MOESM1_ESM.pdf]

Supplementary Information

From Head Micro-Motions Towards CSF Dynamics and Non-invasive Intracranial Pressure Monitoring

Arnošt Mládek[1,2], Václav Gerla[2], Petr Šeba[3], Vladimír Kolář[4], Petr Skalický[1,5], Helen Whitley[1], Lenka Lhotská[2,6], Vladimír Beneš[1], and Ondřej Bradáč[1,5*]

[1]Department of Neurosurgery and Neurooncology, 1st Faculty of Medicine, Charles University in Prague and Military University Hospital, Czech Republic
[2]Department of Cognitive Systems and Neurosciences, Czech Institute of Informatics, Robotics and Cybernetics, Czech Technical University, Prague, Czech Republic
[3]Department of Physics, University of Hradec Králové, Hradec Králové, Czech Republic
[4]Department of Technical Development, LINET spol. s.r.o., Slaný, Czech Republic
[5]Department of Neurosurgery, 2nd Faculty of Medicine, Charles University in Prague and Motol University Hospital, Czech Republic
[6]Department of Natural Sciences, Faculty of Biomedical Engineering, Czech Technical University, Prague, Czech Republic

* corresponding author: ondrej.bradac@uvn.cz

Differential geometry of curves: the underlying theory

From the physical point of view, the reflection process inside the skull does not depend on the head orientation, since the brain structures are fixed with respect to the cranium. This means that the ideal measurement of the head micromotions should be done in a coordinate system firmly connected to the cranium.
Let us have n sensors measuring the head tremble. The signal curve β is a function: $\beta: I \rightarrow \mathfrak{R}^n$ from the time interval $I \in \mathfrak{R}$ to a n-dimensional space $\mathfrak{R}^n$ such that $t \rightarrow (\beta_1(t), \beta_2(t), \ldots, \beta_n(t))$, where $\beta_k(t)$ is the signal obtained by the n-th sensor at time t.
The signal curve β is a geometrical object that clearly results from the physical process inside the skull. Hence it has to be, in line with Newtonian physics, invariant with respect to the Euclidean group (translations and rotations). On the other hand, the projections $\beta_k(t)$ depend on the positions of the measuring sensors with respect to the head. Adjustment of the relative positions, for instance by rotating the head, would result in changes in the projections $\beta_k(t)$ but it cannot change the geometric object β itself. The change in the projections simply means that we observe β from another perspective. The natural question that arises is whether it is possible to completely describe the β curve via some Euclidean group-invariant function of $\beta_k(t)$. The answer is contained in the fundamental theorem of curves which states that for a given smooth n-dimensional curve β there are exactly n-1 functions $(k_1(t), k_2(t), \ldots, k_{n-1}(t))$ that completely determine the curve. These functions are usually denoted as Cartan curvatures. The interesting point is that they can be evaluated from the measured signal projections

(β_1(t), β_2(t), ..., β_n(t)). Once obtained, the functions (k_1(t), k_2(t), ..., k_{n-1}(t)) do not change with the head motion despite being calculated from the head position-dependent projections β_k(t).

In the present study we use only the first function k_1(t) since it can be obtained from the first and second time derivatives of measured β_k(t) projections. The higher functions k_2(t), ..., k_{n-1}(t) contain higher derivatives and are therefore more sensitive to the noise. Additionally, as in the Taylor series, the significance of the terms usually decreases with the term order. Taking only k_1(t) term into account gives us first-order approximation of β. In a two-dimensional case we have only one curvature k_1(t) fully describing the curve and it has a simple meaning: it is the inverse radius of the osculating circle at a given point of the curve (Fig. S1).

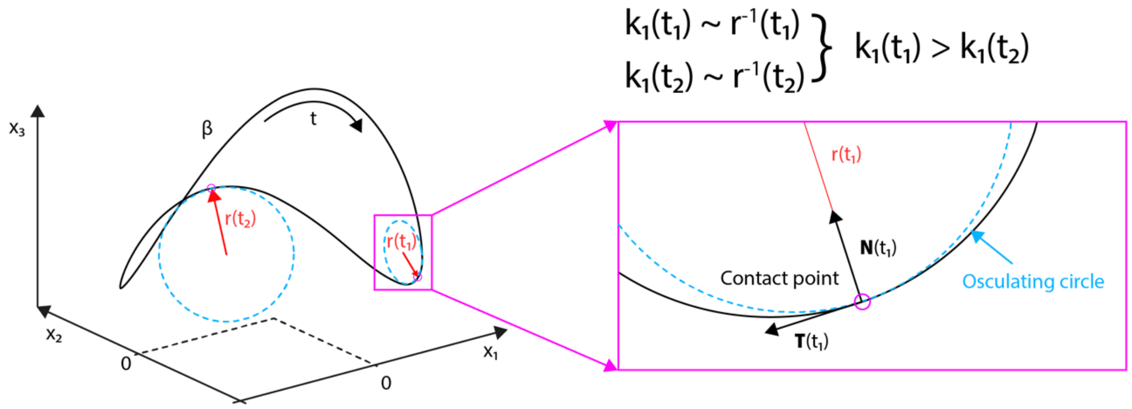

Fig. S1. Example of curvature calculation in 3D space. Osculating circles of a space curve β(t) = (β_1(t), β_2(t), β_3(t)) at time t_1 and t_2. Vector **N**(t_1) is the normal unit vector collinear with the osculating circle (dashed blue line) radius **r**(t_1) (red), **T**(t_1) is the tangent unit vector pointing in the direction of motion and is perpendicular to **N**(t_1). The tangent and the normal vector define the osculating plane at point **r**(t_1). The magnitude of the inverted osculating circle radius 1/|**r** (t)| defines the curvature k_1(t) of the given curve β at the time t. The figure was generated using Adobe Illustrator 2020 (https://www.adobe.com/).
